# Pyrolysis of Table Sugar

**DOI:** 10.1155/2013/172039

**Published:** 2013-10-02

**Authors:** Adnan Bulut, Selhan Karagöz

**Affiliations:** ^1^Chemistry Department, Kırıkkale University, 71450 Kırıkkale, Turkey; ^2^Chemistry Department, Karabük University, 78050 Karabük, Turkey

## Abstract

Table sugars were pyrolyzed at different temperatures (300, 400, and 500°C) in a fixed-bed reactor. The effect of pyrolysis temperature on yields of liquid, solid, and gaseous products was investigated. As expected the yield of liquid products gradually increased and the yield of solid products gradually decreased when the pyrolysis temperature was raised. The yield of liquid products was greatest (52 wt%) at 500°C. The composition of bio-oils extracted with diethyl ether was identified by means of gas chromatography mass spectrometry (GC-MS), nuclear magnetic resonance (^1^H-NMR), and Fourier transform infrared spectroscopy (FTIR). The following compounds were observed in bio-oils produced from the pyrolysis of table sugar at 500°C: 1,4:3,6-dianhydro-**α**-d-glucopyranose, 5-(hydroxymethyl) furfural, 5-acetoxymethyl-2-furaldehyde, and cyclotetradecane liquid product. The relative concentration of 5-(hydroxymethyl) furfural was the highest in bio-oils obtained from pyrolysis of table sugars at 500°C.

## 1. Introduction

Carbohydrates are biopolymers that consist of carbon, hydrogen, and oxygen. They have very important biological functions, and their properties consist of fuels, energy storage, and metabolic intermediates. A disaccharide is a member of carbohydrate family. Disaccharides are classified as reducing disaccharides and nonreducing disaccharides [1]. If a disaccharide contains a reactive hemiacetal center, it is a reducing disaccharide. The reducing disaccharides are maltose, isomaltose, cellobiose, gentiobiose, xylobiose, mannobiose, and lactose. The nonreducing disaccharides are sucrose and trehalose. The substance known by the term “table sugar” is a disaccharide, which is composed of the bonding of a glucose molecule with a molecule of fructose. Table sugar, which is also known as edible sugar, saccharose, and sucrose, is produced from the extraction of sugar beets and sugar cane. 

Production of bio-oils via pyrolysis of agricultural byproducts has been investigated extensively [2–4]. Sugar cane bagasse was thermochemically converted into liquid, solid, and gaseous products via slow pyrolysis and vacuum pyrolysis [5]. It was reported that the vacuum pyrolysis produced a higher BET specific surface area of char, whereas slow pyrolysis led to an increase in the heating values of char. No information on characterization of liquid products was provided in that study [5]. The bio-oils from vacuum pyrolysis of sugarcane bagasse were extracted using various solvents having different polarities [6]. It was reported that the bio-oils contained various oxygenated hydrocarbons. Fast pyrolysis was applied to sweet sorghum and sweet sorghum bagasse at different temperatures (from 400 to 560°C) [7]. Liquid compositions from the two sorghum feedstocks were found to be similar and the majority of the liquid products were hydroxyacetaldehyde and pyrolytic lignin.

In order to understand the thermal decomposition behavior of materials that contain sugar, it is necessary to do fundamental research on the pyrolysis of sugars. In the current investigation, the pyrolysis of table sugar was performed at 300, 400, and 500°C. The liquid products were identified by applying analytical methods (GC-MS, FTIR, and ^1^H-NMR).

## 2. Materials and Methods

### 2.1. Material

The table sugar used in this study was in the form of crystalline powder and taken from a local market in Safranbolu, Turkey. Diethyl ether was purchased from Carlo Erba. Anhydrous sodium sulfate was purchased from Sigma Aldrich.

### 2.2. Pyrolysis Procedure

Pyrolysis experiments were performed in a batch reactor at 300, 400, and 500°C for 1 h under continuous nitrogen gas flow (30 mL min^−1^). Details about the pyrolysis procedure can be found in a previous report [2]. Briefly, 50 g of table sugar was placed into the reactor. The system was heated to the desired temperatures (300, 400, and 500°C) at a heating rate of 5°C. The pyrolysis experiments were repeated three times, and an average standard deviation was found to be 2.5 wt% for liquid and solid yields.

### 2.3. Analysis Procedure

Bio-oils were recovered from liquid products with diethyl ether extraction. The obtained diethyl ether fraction was dried over anhydrous sodium sulfate. The bio-oil was obtained after removing solvent (ether). The bio-oil was analyzed by GC-MS (6890 Gas Chromatograph Agilent; 30 m × 0.25 mm i.d. phenyl methyl siloxane capillary column HP-5MS). The following oven temperature program was used in the experiment: the oven was initially set to 40°C with a holding time of 10 min; 40–170°C at a rate of 3°C; 5 min holding time; 170–270°C at a rate of 4°C; 10 min holding time; and then 270–300°C at a rate 12°C, 10 min holding time.

 The ^1^H NMR spectrum was recorded in CDCl_3_ on Bruker Spectrospin Avance DPX-400 Spectrometer. Chemical shifts were given in ppm downfield from tetramethylsilane. A Fourier transform infrared (FTIR) spectrum of the selected bio-oil was recorded using a Jasco FT-IR 480.

## 3. Results and Discussions

**Figure 1:**
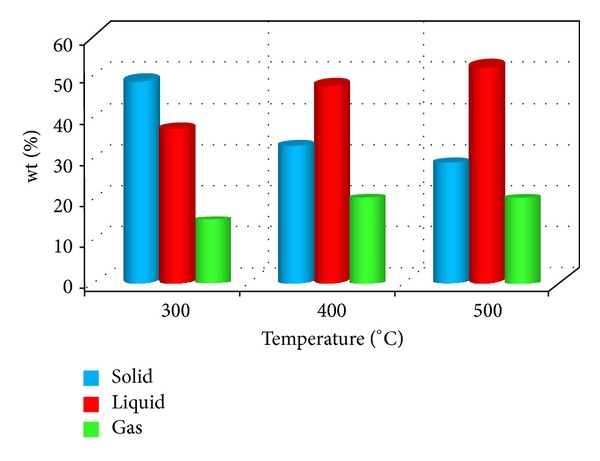
Product distributions from the pyrolysis of table sugar at the temperatures of 300, 400, and 500°C.

Yields of liquid, solid, and gaseous products from the pyrolysis of table sugar at 300, 400, and 500°C are shown in Figure 1. The solid residue yields were 49 and 33 wt% at 300°C and 400°C, respectively. After a further rise in the temperature to 500°C, the solid residue was reduced to 28.5 wt%. The liquid yields were 37 and 48 wt% at 300 and 400°C, respectively. The highest liquid yield was obtained at 500°C, and it was 52 wt%. The lowest gas yield was 14 wt%, and it was obtained at 300°C as expected. After the increase in the temperature from 300 to 400°C, the gas yield increased and was found to be 19 wt%. An increase in pyrolysis temperature from 400 to 500°C did not change the yield of gas product (~19 wt%).

The identification of compounds in the ether extract from the pyrolysis of table sugar at 500°C is presented in Table 1. The identified compounds were 1,4:3,6-dianhydro-*α*-d-glucopyranose, 5-(hydroxymethyl) furfural, 5-acetoxymethyl-2-furaldehyde, and cyclotetradecane. The relative concentration of 5-(hydroxymethyl) furfural (HMF) was the highest among the others. As is well known, HMF is produced from the dehydration of sugars upon heat treatment [8, 9]. HMF is also a very important intermediate for the production of the biofuel dimethylfuran (DMF), as well as for the production of other molecules such as levulinic acid, 2,5-furandicarboxylic acid (FDA), 2,5-diformylfuran (DFF), dihydroxymethylfuran, and 5-hydroxy-4-keto-2-pentenoic acid [10]. 1,4:3,6-dianhydro-*α*-d-glucopyranose was also observed. Analysis of extracted and volatile components in blackstrap molasses feed was completed [11]. It was reported that hexane and diethyl ether extracts of blackstrap molasses contained a large variety of compounds. One of the identified compounds in the ether extract was 1,4:3,6-dianhydro-*α*-d-glucopyranose. The bio-oil obtained from the pyrolysis of food-processing sewage sludges was analyzed and reported [12]. Analysis showed that the identified compounds were mainly nitrogen and/or oxygen containing compounds in biosludges from the fructose-manufacturing factor. One of the identified compounds at 500°C in the liquid product from biosludges of fructose-manufacturing factor was cyclotetradecane. The formation of HMF, 1,4:3,6-dianhydro-*α*-d-glucopyranose, and cyclotetradecane from the pyrolysis of table sugar is in agreement with these previous reports [8, 9, 11, 12]. 

**Figure 2:**
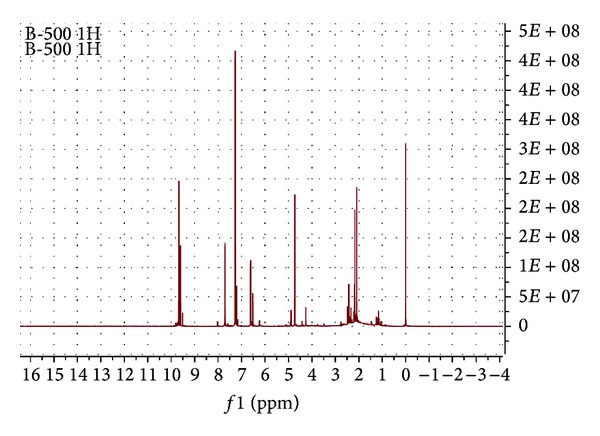
^1^H NMR spectrum of the ether extract obtained from the pyrolysis of table sugar at 500°C.

Figure 2 shows the ^1^H NMR spectrum of the ether extract produced from the pyrolysis of the table sugar at 500°C. As expected, aldehyde protons appeared around 9.5 ppm. Two aldehyde signals prove that there are two furfural groups. Aromatic hydrogens belonging to furfurals shifted to ~7.75, 7.25, 6.7, and 6.5 ppm down fields. Methylene bonded to furfural, and the hydroxyl group signal was seen around 4.75 ppm downfield due to the high electronegativity of oxygen. The sharp signal at 7.27 ppm came from the solvent (CDCl_3_).

**Figure 3:**
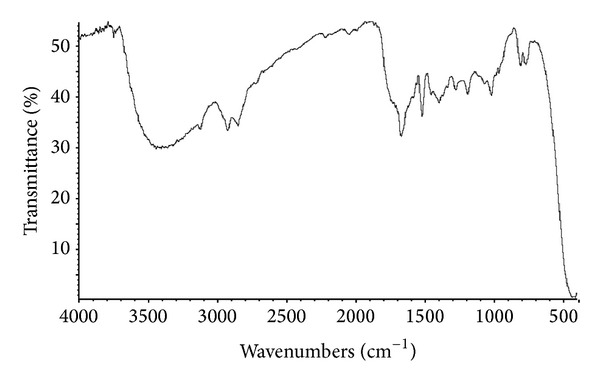
FTIR spectrum of the ether extract produced from the pyrolysis of table sugar at 500°C.

 Functional group analysis of the ether extract obtained from the pyrolysis of table sugar at 500°C was completed by FTIR spectrometry. The FTIR spectrum of the ether extract is presented in Figure 3. The broad band between 3000 and 3500 cm^−1^ is due to the hydroxyl group. The peak at 1683 cm^−1^ is assigned to the carbonyl group stretching vibration, which indicates the presence of aldehydes groups. ^1^H NMR spectrum and FTIR spectrum of the ether extract from the pyrolysis of the table sugar at 500°C supports the results of the GC-MS analysis.

## 4. Conclusion

Pyrolysis of table sugar was performed at 300, 400, and 500°C for 1 h. The increase in temperature resulted in an increase in liquid product yields and a decrease in solid residue yields. The highest liquid yield was found to be 52 wt%, and it was obtained at 500°C. The bio-oil from the extraction of the liquid product contained mainly HMF together with minor compounds: 1,4:3,6-dianhydro-*α*-d-glucopyranose, 5-acetoxymethyl-2-furaldehyde, and cyclotetradecane. The results from ^1^H NMR and FTIR are in agreement with GC-MS results.

## Figures and Tables

**Table 1:** Identified compounds in ether extract from the pyrolysis of table sugar at 500°C.

Retention time	Quality	Identified compounds	MS ions (*m*/*z*)	Peak area, %
24.58	91	1,4:3,6-Dianhydro-*α*-d-glucopyranose	57, 69, 73, 86, 98, 114, 144	0.71
25.82	94	5-(Hydroxymethyl)furfural	53, 69, 81, 97, 109, 126	93.77
29.35	83	5-Acetoxymethyl-2-furaldehyde	53, 69, 79, 97, 109, 126	1.17
38.37	97	Cyclotetradecane	55, 69, 83, 97, 111, 125, 139, 196	0.89
